# Italian pediatric respiratory society recommendations on pediatric pulmonary function testing during COVID-19 pandemic

**DOI:** 10.1186/s13052-020-00829-0

**Published:** 2020-05-24

**Authors:** Elisabetta Bignamini, Salvatore Cazzato, Renato Cutrera, Giuliana Ferrante, Stefania La Grutta, Amelia Licari, Enrico Lombardi, Fabio Midulla, Giorgio Piacentini, Massimo Pifferi, Francesca Santamaria, Giancarlo Tancredi, Attilio Turchetta, Italian Pediatric Respiratory Society (IPRS) Committee Members

**Affiliations:** 1 Pediatric Pulmonology, Cystic Fibrosis Centre, AOU Città della Salute e della Scienza, Torino, Italy; 2 Department of Mother and Child Health, Salesi Children’s Hospital, Ancona, Italy; 3 Department of Pediatrics, Bambino Gesù Children’s Hospital, Rome, Italy; 4 Department of Health Promotion Sciences, Maternal and Infant Care, Internal Medicine and Medical Specialities, University of Palermo, Palermo, Italy; 5 Institute for Biomedical Research and Innovation (IRIB), National Research Council (CNR), Palermo, Italy; 6 Pediatric Clinic, Fondazione IRCCS Policlinico San Matteo, University of Pavia, Pavia, Italy; 7 Pediatric Pulmonary Unit, Meyer Pediatric University Hospital, Florence, Italy; 8 Department of Pediatrics, “Sapienza” University of Rome, Rome, Italy; 9 Department of Surgical Sciences, Dentistry, Gynaecology and Paediatrics, University of Verona, Verona, Italy; 10 Department of Paediatrics, University Hospital of Pisa, Pisa, Italy; 11 Department of Translational Medical Sciences, Pediatric Pulmonology, Federico II, Naples, Italy; 12 Sport Medicine Unit, Bambino Gesù Children’s Hospital, Rome, Italy

**Keywords:** COVID-19, SARS-CoV-2, Children, Pulmonary function testing

## Abstract

**Background:**

Effective prevention and control strategies are mandatory to prevent SARS-CoV-2 infection.

**Main text:**

The Italian Pediatric Respiratory Society promotes a series of new recommendations that should be followed in pulmonary function testing laboratories during the COVID-19 pandemic.

**Conclusion:**

Pulmonary function testing should be performed in children with chronic lung disease only if it is needed to guide management and limited to the necessary tests, namely spirometry. When performed, strict infection control measures should be followed due to the potential risk of transmitting SARS-CoV-2.

## Main text

The coronavirus disease 2019 (COVID-19) is a public health emergency of international concern caused by a newly discovered coronavirus (severe acute respiratory syndrome coronavirus 2, SARS-CoV-2) [1]. Person-to-person transmission of SARS-CoV-2 occurs primarily through close contact with an infected person, mainly occurring via respiratory droplets and after touching contaminated objects [1]. Accordingly, pulmonary function testing (PFT) could be considered at high-risk for viral transmission due to the potential for coughing and droplet formation during PFT procedures [2]. The most likely surfaces for possible contamination by this route are mouthpieces, proximal valves, and tubing. Also, both technical and clinical staff are usually in close contact with the patient during PFT for achieving optimal cooperation and best results [2].

Effective prevention and control strategies are mandatory to prevent SARS-CoV-2 infection in the PFT laboratories.

The Italian Pediatric Respiratory Society (IPRS) promotes a series of new recommendations that should be followed by healthcare workers in PFT laboratories during the COVID-19 pandemic.

1. Routine PFT should not be performed to reduce the risk of viral transmission. When necessary, PFT should be limited to the essential tests, namely spirometry. Spirometry should be considered essential only if therapeutic decisions are urgent in children with chronic lung disease (cystic fibrosis, primary ciliary dyskinesia, severe uncontrolled asthma, lung transplantation, onco-haematological diseases before and after hematopoietic stem cell transplantation), following adequate infection control measures. Outside these indications, PFT should only be performed if required by the pediatric pulmonologist since some individual circumstances may be unique or require contextual consideration. Decisions of performing PFT should be balanced between the potential risks and the need for PFT to make any therapeutic decision [2].
2. It is strongly recommended not to perform PFT in confirmed or suspected cases of COVID-19. PFT should be postponed after the resolution of symptoms and two negative real-time Polymerase Chain Reaction (RT-PCR) tests on nasopharynx and throat swabs for SARS-CoV-2 performed at 24-h interval [3].
3. Appropriate procedures and measures during the COVID-19 pandemic should be put in place in order to target 1) clinical and technical staff of PFT laboratory; 2) children and their caregivers; 3) PFT machines and devices; 4) environmental cleaning and disinfection.
4. The clinical and technical staff in the PFT laboratory are required to adhere to the World Health Organization (WHO) technical document that highlights the rights and responsibilities of healthcare professionals [4]. A specific regulatory protocol has been signed on March 14th, 2020 by unions and companies in agreement with the Italian government to protect the health and safety of workers from possible infection with SARS-CoV-2 and ensure the safety of the work environment [5]. This regulatory protocol should be adopted within all workplaces, including hospital facilities, in addition to the provisions of the Italian Prime Minister’s Decree of March 11th, 2020 [6].
5. Beyond community prevention measures that comply with the WHO guidelines [7], additional measures are necessary for healthcare professionals who perform PFT in order to preserve themselves and prevent the spreading of the virus in the health community. At this purpose, it is recommended to follow the indications delivered by the Italian “Istituto Superiore di Sanità” (ISS) Working Group on Infection Prevention and Control [8] concerning put on, use, take off and dispose of personal protective equipment, such as masks, disposable gowns, gloves, goggles or face shields, in the specific work context and job carried out.
6. All healthcare professionals should be adequately trained and updated on the risks of occupational exposure and the available prevention and protection measures. They should follow established occupational safety and health procedures, and avoid exposing themselves and others to health and safety risks. Recommendations on maintaining hygiene in a PFT laboratory are particularly relevant for protecting patients and healthcare professionals and for providing a safe and work environment [4].
7. Patients’ access (including any single accompanying person) to the areas dedicated to performing PFT should be allowed after having checked the absence of fever (body temperature < 37.5 °C) and any sign/symptom of respiratory disease (e.g., cough, shortness of breath). Patients and any single accompanying person will be asked to sanitize their hands with a hydro-alcoholic solution (or with soap and water) for at least 30 s and wear a surgical mask. Immunocompromised patients should be scheduled for testing at the start of the day, before other patients arrive, whereas infected patients should be scheduled for testing at the end of the day to prevent cross-infection. Patients/subjects should be isolated in a separate area for testing to minimize contacts with potentially infectious patients. It is very important that patients who present with acute respiratory symptoms wear a surgical mask. When applicable, a distance of at least one meter from the patient should be maintained. Measures for COVID-19 prevention and control should be engaged in addition to standard measures for preventing and controlling cross-infection acquired from the PFT laboratory.
8. Implementing hygiene control rules (handwashing, use of gloves for handling the mouthpieces or internal surfaces of the spirometer) prevents the spread of infections from the patient to healthy subjects and healthcare professionals. Sources of cross-infection in a PFT laboratory include direct contact, aerosolized particles, saliva, and skin contact. Mouthpieces have the highest risk of contamination since they come in direct contact with the patient. Mouthpieces need to be replaced after every use to minimize the risk of cross-infection. Overall, to perform PFT, disposable materials (filters, mouthpieces, nose clips, flow sensors) should be discarded after a single use. Where reusable items are utilized, they should be managed carefully and should be thoroughly cleaned and disinfected as recommended by manufacturers and local infection control policy.
9. We recommend enhanced cleaning and disinfection procedures for both the PFT laboratory and equipment. The testing space and equipment should be cleaned with chlorine solutions at a concentration of 1000 ppM or with polyphenol solution at a concentration of 1%, according to the manufacturer’s indications. Biological waste should be handled appropriately and disposed to reduce the risk of cross-infection. Separation of infectious and noninfectious waste help to control infection. Solid infectious waste should be discarded in containers labelled with the universal symbol of biological hazards. Routine cleaning of floors with detergent and water is recommended as dust and dirt may neutralize the action of disinfectant or sterilant. Therefore, disinfection should always be undertaken with a detergent cleaner [9].
10. Pressurized metered-dose inhaler (pMDI) via a spacer should be considered the preferred device for the administration of any drugs (e.g., salbutamol). The use of nebulizers should be avoided to decrease the risk of disseminating COVID-19 to other patients and to physicians, nurses and other personnel in the laboratory, unless an airborne infection isolation room is available [10]. Spacers should not be shared among patients.

## Conclusions

COVID-19 is an emerging, rapidly evolving situation, and the IPRS is following developments closely.

During the COVID-19 containment phase, the IPRS recommends avoiding routine PFT and limiting to the necessary tests, namely spirometry. PFT should be performed in children with chronic lung disease only if it is needed to guide management and if comprehensive infection control can be maintained in the PFT laboratories. When performing PFT, strict infection control measures should be followed.

## Data Availability

Not applicable.

## Abbreviations

COVID-19: Coronavirus disease 2019
ISS: Istituto Superiore di Sanità
PFT: Pulmonary function testing
p-MDI: pressurized metered-dose inhaler
RT-PCR: Real-time Polymerase Chain Reaction
SARS-CoV-2: Severe acute respiratory syndrome coronavirus 2
IPRS: Italian Pediatric Respiratory Society
WHO: World Health Organization
